# Performance comparison of III–V//Si and III–V//InGaAs multi-junction solar cells fabricated by the combination of mechanical stacking and wire bonding

**DOI:** 10.1038/s41598-019-40727-y

**Published:** 2019-03-13

**Authors:** Yu-Cheng Kao, Hao-Ming Chou, Shun-Chieh Hsu, Albert Lin, Chien-Chung Lin, Zun-Hao Shih, Chun-Ling Chang, Hwen-Fen Hong, Ray-Hua Horng

**Affiliations:** 1 Graduate Institute of Precision Engineering, National Chung Hsing University, Taichung, 40227 Taiwan Republic of China; 2 Institute of Electronics, National Chiao Tung University, Hsinchu, 30010 Taiwan Republic of China; 3 Institute of Photonic System, National Chiao Tung University, Tainan, 71150 Taiwan Republic of China; 4 Institute of Nuclear Energy Research (INER), Atomic Energy Council, Executive Yuan, Taoyuan, 32546 Taiwan Republic of China; 5 Center for Emergent Functional Matter Science, National Chiao Tung University, Hsinchu, 300 Taiwan Republic of China

## Abstract

The integration of III–V and Si multi-junction solar cells as photovoltaic devices has been studied in order to achieve high photovoltaic conversion efficiency. However, large differences in the coefficients of thermal expansion and the lattice parameters of GaAs, Si, and InGaAs have made it difficult to obtain high-efficiency solar cells grown as epilayers on Si and InP substrates. In this paper, two types of devices, including GaInP/GaAs stacked on Si (GaInP/GaAs//Si) and GaInP/GaAs stacked on InGaAs (GaInP/GaAs//InGaAs), are fabricated via mechanical stacking and wire bonding technologies. Mechanically stacked GaInP/GaAs//Si and GaInP/GaAs//InGaAs triple-junction solar cells are prepared via glue bonding. Current-voltage measurements of the two samples are made at room temperature. The short-circuit current densities of the GaInP/GaAs//Si and GaInP/GaAs//InGaAs solar cells are 13.37 and 13.66 mA/cm^2^, while the open-circuit voltages of these two samples are measured to be 2.71 and 2.52 V, respectively. After bonding the GaInP/GaAs dual-junction with the Si and InGaAs solar cells, the conversion efficiency is relatively improved by 32.6% and 30.9%, respectively, compared to the efficiency of the GaInP/GaAs dual-junction solar cell alone. This study demonstrates the high potential of combining mechanical stacked with wire bonding and ITO films to achieve high conversion efficiency in solar cells with three or more junctions.

## Introduction

It is well known from theoretical simulation results that tandem-type III–V material multi-junction (with six junctions) solar cells have higher conversion efficiency than solar cells constructed using other materials^1,2^. However, it is difficult to obtain six-junction III–V solar cells via epitaxial growth, owing to limitations in lattice matching^2,3^. Therefore, improving the conversion efficiency of multi-junction solar cells via non-epitaxial processes has been widely investigated to achieve high performance^4–6^. Moreover, new types of surface management, anti-reflection coating layers, and electrode fabrication techniques have been employed to improve the electric current extraction and to increase the amount of incident light entering the absorption region of solar cells^7–13^. Although a high conversion efficiency of over 30% under the one-sun air mass 1.5 (AM1.5G) spectrum condition has been achieved using GaInP/GaAs/Ge triple-junction (TJ) solar cells^14^, the cost of such a device is high because the underlying Ge substrate is very expensive. On the other hand, the efficiency of a solar cell up to 43.5% at 306 suns under the AM1.5 spectrum has been reported using inverted metamorphic GaInP/GaAs/In_0.3_Ga_0.7_As solar cells^15^. However, the inverted metamorphic structure suffers from lattice mismatch issues for these epilayers.

In recent years, the fabrication of III–V compound dual-junction (DJ) solar cells on silicon to form TJ solar cells has been reported^16^. However, direct growth of 1.9-eV GaInP solar cells on Si using metal–organic vapor phase epitaxy (MOVPE) is challenging^17^ because of large differences between the thermal expansion coefficients^17^ and a 4% lattice mismatch^17^ between Si and the most common III–V layers that are lattice-matched to GaAs.

Thus, multi-junction solar cells are instead fabricated using the mechanical bonding method^4–6^. The mechanical stacking technologies that can be used to fabricate multi-junction solar cells include glue-, metal-, and fusion-bonding^18–22^. In general, the glue-bonding method is less expensive and the bonding temperature is low (<250 °C). However, in the metal-bonding method, the bonding temperature is dependent on the eutectic temperature. Meanwhile, the fusion-bonding method always requires a high vacuum system and/or a high bonding temperature (>400 °C).

It is worth mentioning that even a stacked multi-junction solar cell can be successfully fabricated and the short-circuit current is always smaller compared with that before bonding. Moreover, it has been reported that transparent conductive oxides (TCO) can be applied as electrodes in solar cells^12,23–28^. TCO electrodes not only form an ohmic contact with the top contact layer of the solar cell, but also enhance the performance of solar cells thanks to the highly transparent layer.

In this study, we present a new fabrication method using a combination of mechanical stacking of the monolithic DJ solar cells with ITO electrodes and a third solar cell, followed by wire bonding. The advantages of combining mechanical stacking and wire bonding are that complex epitaxial structures are not required, low fabrication cost and enhancing the performance of solar cells by the intermediate transparent electrode. It has the potential to produce high-efficiency multi-junction solar cells with absorption wavelengths longer than that of monolithic solar cells. According to the proposed method, the Ga_0.51_In_0.49_P/GaAs (a lattice-matched epitaxial growth system with a tunnel junction) and In_0.53_Ga_0.47_As device structures are grown on GaAs and InP substrates, respectively. Then, the GaInP/GaAs solar cell, Si solar cell, and InGaAs solar cell are bonded via mechanical stacking and wire bonding to form multi-junction solar cells. The fabrication processes and optoelectronic performances, particularly for the current-matching issues of the resulting solar cells, are discussed in detail. These designs of solar cells are also analyzed through simulation and equivalent circuits.

## Results and Discussion

**Figure 1:**
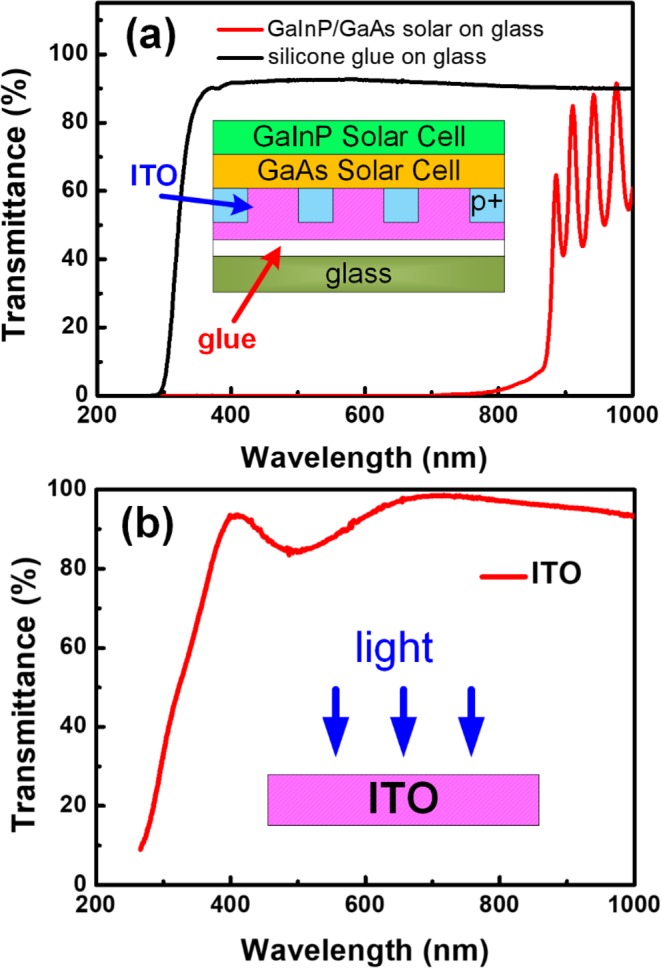
Transmittance spectra of (**a**) a GaInP/GaAs solar cell prepared on a glass and silicone glue on glass substrate and (**b**) only an ITO film in the wavelength range of 200–1000 nm. A schematic of the DJ solar cell and transmittance data of ITO are shown in the insets of (**a**,**b**), respectively.

To fabricate tandem solar cells via mechanical stacking, it is important to evaluate the absorption characteristics of the thin DJs with transparent ITO electrodes and transfer them onto glass substrates and the bonding glue. The transmittance spectra of GaInP/GaAs/ITO/glue/glass and the silicone glue on glass substrate in a wavelength range of 200–1000 nm were measured and shown in Fig. 1(a). For the transmittance spectrum of GaInP/GaAs/ITO/glue/glass, it was found that the transmittance increased gradually at wavelengths greater than 780 nm. At wavelengths of 800–900 nm, the transmittance of GaInP/GaAs/ITO/glass increased sharply from 1% to 65%. Furthermore, when the wavelength was increased from 880 to 1000 nm, the GaInP/GaAs/ITO cell device exhibited a higher transmittance of 60–80%. As concerning the bonding silicone glue, used to bond GaInP/GaAs dual-junction solar epilayer to glass, it is widely used for LEDs and solar cells packaged with high transmission of wavelength, heat tolerance, photostability and resistance to ultraviolet discoloration. The transmittance of silicone/glass is about 85% at the 350 nm wavelengths as shown in Fig. 1(a). Furthermore, when the wavelength was increased from 400 to 1000 nm, the silicone binder exhibited a transmittance of 90%. In this study, the silicone layer was below the III-V DJ. The III-V DJ has effectively absorbed UV light. It means that the silicone glue can not be degraded by the UV light and presents stability and high transparency for the wavelength longer than 350 nm. Furthermore, the transmittance of ITO is also important, owing to the embedding of ITO between the DJ and the bottom cell in this structure. Thus, the ITO was deposited on the glass substrate which is used to analysis transmittance. The transmittance of the ITO-only film can be obtained by the calculation of T (ITO/glass)/T (glass). The transmittance of the ITO-only film from 880 to 1000 nm is above 90%, as shown in Fig. 1(b). This means some of light would be reflected or scattered by these different epilayers and optical layers. As concerning the light distribution from the III-V epilayer toward the bottom cells, it has been simulated and shown in the supplementary information. Nevertheless, the 60–80% light from 880 to 1000 nm can be utilized by the bottom cell. In addition, an oscillation was formed in the transmittance spectrum, and it resulted from constructive and destructive interferences of light with these materials. The results confirm that the ITO film has good potential to be used as the back-side electrode in solar cells, and it can effectively replace the conventional opaque metal. Moreover, this result suggests that DJ GaInP/GaAs solar cells absorb light only with a wavelength of 300–880 nm. To effectively use sunlight, Si and InGaAs solar cells were fabricated to absorb light waves of longer wavelengths (>880 nm).

**Figure 2:**
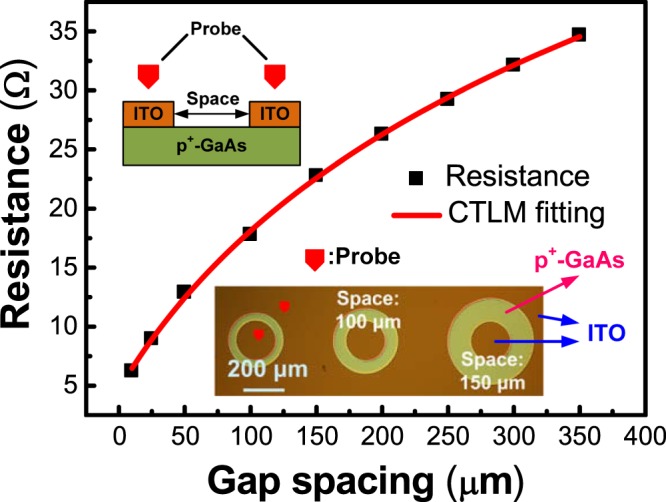
Contact resistance between the ITO film and p^+^-GaAs layer. The top view image of CLTM contact pads with the spacings of 100 and 150 μm is shown in the lower inset. Schematics of p^+^-GaAs layer with ITO contact layer is shown in the upper inset.

The contact characteristics between the ITO and the p^+^-GaAs layer were another important issue in estimating the practicability of a back-side electrode in III–V compound solar cells. Here, the conductivity of ITO is 1.8 × 10^−4^ Ω·cm. Figure 2 shows the result of a CTLM measurement of the contact resistance for the gap spacing between the ITO film and the p^+^-GaAs layer. It was measured using CTLM patterns with nine circular contact pads (diameter 200 μm) and separated by designed spacings of 10, 25, 50, 100, 150, 200, 250, 300, and 350 μm. The measured resistance values were dependent on the gap spacings (10–350 μm) of the contact pads. The lower inset of Fig. 2 is the top view image of CTLM contact pads with the spacing of 100 and 150 μm. The schematics of p^+^-GaAs layer with ITO contact layer is shown in the upper inset. Good ohmic contact behavior was found between the ITO film and the p^+^-GaAs layer without thermal annealing.

Notably, AuBe/Au back-side metal electrodes are used commonly in the traditional solar cell structure, and thermal annealing at approximately 400 °C is necessary. This type of metal will limit the device performance in terms of mechanical stacking of solar cells because of its opacity. Thus, the as-coated ITO film was used as the transparent conductive layer for solar cell applications in this work. By fitting these resistance values^29^, the contact resistance between ITO and p^+^-GaAs was estimated to be 1.62 × 10^−4^ Ω-cm^2^. This shows that the contact resistance between ITO and p^+^-GaAs is sufficient for III–V compound solar cells. Moreover, the deposition of the ITO film on the p^+^-GaAs layer enables the use of ITO films as the back-side electrode of solar cells.

**Figure 3:**
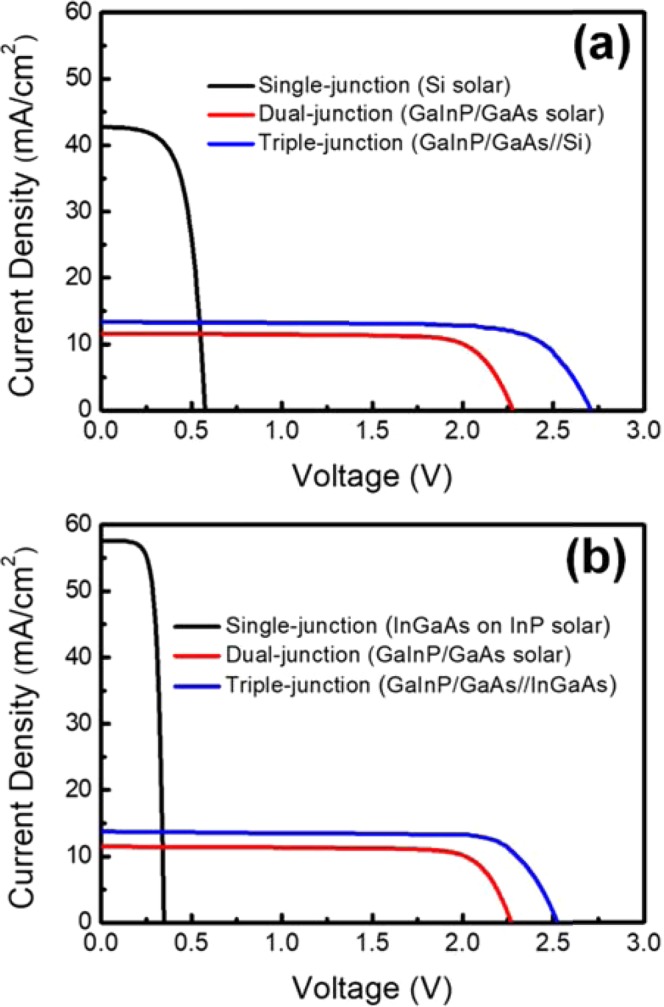
J–V characteristics of (**a**) GaInP/GaAs//Si and (**b**) GaInP/GaAs// InGaAs TJ solar cells measured using a one-sun AM1.5G light source. They were measured under the individually fabricated dual-, single-, and stacked junctions.

**Figure 4:**
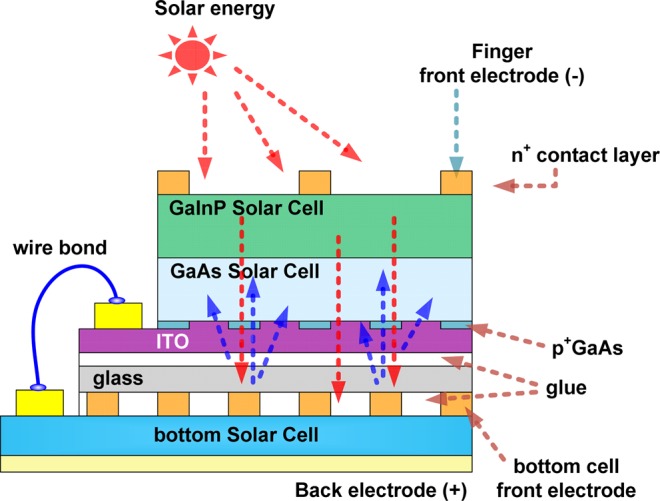
Operation mechanism of the light reflection path.

Figure 3 shows the measured J–V characteristics of Si, InGaAs, and GaInP/GaAs DJ and TJ solar cells under the one-sun condition in the AM1.5G solar simulator. The TJ solar cells with two structures, including the GaInP/GaAs//Si (Fig. 3(a)) and GaInP/GaAs//InGaAs (Fig. 3(b)) multi-junction solar cells, were fabricated via mechanical stacking. The detailed cell device characteristics, including open-circuit voltage (V_oc_), short-circuit current density (J_sc_), fill factor (FF), and conversion efficiency (η) of the two samples are given in Tables 1 and 2. The Si-based V_oc_ values of the Si and the GaInP/GaAs DJ and TJ solar cells were 0.57, 2.28, and 2.71 V, respectively. Additionally, the J_sc_ values were 42.78, 11.59, and 13.37 mA/cm^2^, respectively. Moreover, the conversion efficiencies of the Si and the GaInP/GaAs DJ and TJ solar cells were 15.50%, 20.60%, and 27.31%, respectively. The InGaAs-based V_oc_ values of the InGaAs, GaInP/GaAs DJ and TJ solar cells were found to be 0.35, 2.27, and 2.52 V, respectively. The J_sc_ densities were 57.65, 11.44, and 13.66 mA/cm^2^, respectively. The conversion efficiencies of the InGaAs and GaInP/GaAs DJ and TJ solar cells were 14.37%, 20.58%, and 26.95%, respectively. It is worth mentioning that the J_sc_ increased after the mechanical stacking, regardless of whether GaInP/GaAs was stacked on Si or InGaAs solar cells. This could be due to light with a shorter wavelength not being absorbed completely. This light penetrated the ITO/glue/glass/glue and then was reflected by the top-electrodes of the bottom cell toward the top cell. The light path is shown in Fig. 4. Light distribution for the light penetrated the ITO/glue/glass/glue was shown in the supplementary information. In this study, the top surface of the Si solar cell had been roughened, making the surface of InGaAs smoother than that of the Si solar cell. This means that the top-electrode reflectivity of InGaAs is higher than that of Si. The increase in J_sc_ (a relative improvement of 19.4%) of GaInP/GaAs//InGaAs is more obvious than that (15.3%) of GaInP/GaAs//Si. After stacking or wafer bonding, the J_sc_ of the multi-junction solar cells is always reduced as compared with that of the top cell before bonding. Using the structure in this study, the J_sc_ can be increased after mechanical stacking. The light reflected toward the top absorber layer was demonstrated in our previous study^30^.

**Table 1:** Electrical properties of GaInP/GaAs//Si TJ solar cells with various electrode structures obtained from J–V curve.

Si type	V_oc_(V)	J_sc_ (mA/cm^2^)	FF (%)	η (%)
Single-junction Si solar cell	0.57	42.78	63.63	15.50
Dual-junction solar cell	2.28	11.59	77.98	20.60
Triple-junction solar cell	2.71	13.37	75.40	27.31

V_oc_: open-circuit voltage; J_sc_: short-circuit current density; FF: fill factor; η: efficiency.

**Table 2:** Electrical properties of GaInP/GaAs/InGaAs TJ solar cells with various electrode structures obtained from J–V curve.

InGaAs type	V_oc_(V)	J_sc_ (mA/cm^2^)	FF (%)	η (%)
Single-junction InGaAs solar cell	0.35	57.65	71.24	14.37
Dual-junction solar cell	2.27	11.44	79.25	20.58
Triple-junction solar cell	2.52	13.66	78.30	26.95

V_oc_: open-circuit voltage; J_sc_: short-circuit current density; FF: fill factor; η: efficiency.

Nevertheless, as compared with the efficiencies of the DJ solar cells, the conversion efficiencies of the Si-based and InGaAs-based TJ solar cells were higher by 32.6% and 30.9%, respectively. The V_oc_ of the Si solar cells is larger than that of the InGaAs solar cells. However, the J_sc_ of the Si solar cell was smaller than that of the InGaAs solar cells. Obviously, an increase in V_oc_ could contribute more to the efficiency of a TJ solar cell than an increase in J_sc_. This also shows that wire bonding and mechanical stacking are useful for increasing the conversion efficiency of multi-junction solar cells.

**Figure 5:**
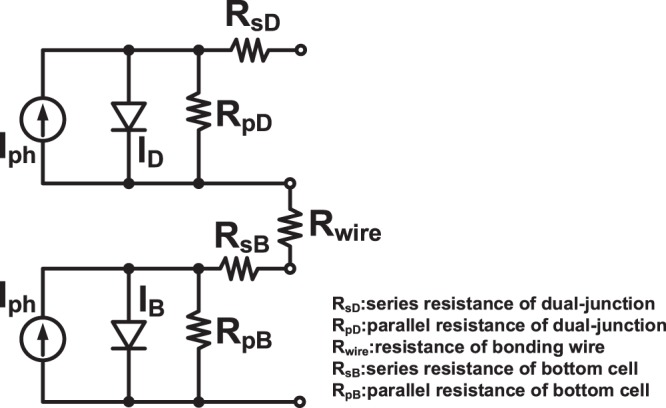
Equivalent circuit of a mechanically stacked TJ solar cell.

On the other hand, the series resistance (R_s_) and parallel resistance (R_p_) can be calculated using Fig. 3 and are shown in Table 3. The values of R_p_ for these solar cells were determined from the slope of the I–V curve near the short-circuit current point. The value of R_s_ can be obtained according to the following equation^31^:

$${R}_{S}=\frac{nKT}{q}\frac{1}{{I}_{2}-{I}_{1}}\,\mathrm{ln}[\frac{{I}_{SC}-{I}_{2}}{{I}_{SC}-{I}_{1}}]-(\frac{{V}_{2}-{V}_{1}}{{I}_{2}-{I}_{1}})$$

where *n* is an ideality factor and approximately 1.5; T is the cell temperature; *K* is Boltzmann’s constant; and *q* is the elementary electric charge; *I*_*SC*_ is the short-circuit current; and *I*_1_, *I*_2_, *V*_1_, and *V*_2_ are the current densities and voltages of two points on the *I–V* curve. Before the mechanical stacking, the R_s_ and R_p_ values are 647 Ω and 5274 Ω, respectively, for the DJ and 42 Ω and 2109 Ω, respectively, for the InGaAs. After bonding, the Rs and R_p_ values of GaInP/GaAs//InGaAs are 983 Ω and 4040 Ω, respectively. Correspondently, the R_s_ and R_p_ values are 726 Ω and 6000 Ω, respectively, for the DJ and 31 Ω and 380 Ω respectively, for Si. After bonding, the R_s_ and R_p_ values of the GaInP/GaAs//Si solar cell are 763 Ω and 5423 Ω, respectively. The equivalent circuit is shown in Fig. 5. For the stacked solar cell, the R_s_ is equal to R_sD_ + R_wire_ + R_sB_. Here, R_sD_, R_pD_, R_sB_, and R_pB_ are the series and parallel resistances of the DJ solar cell and the series and parallel resistances of the bottom solar cell, respectively. Moreover, there exists the resistance (R_wire_) of the bonding wire due to the mechanic stacking. From the circuit analysis, it is obvious that the series resistance will increase slightly for the TJ solar cell, because the connection of the DJ and bottom cells is by wire bonding. The series resistance increases for both types of TJ solar cells. It was found that the series resistance of GaInP/GaAs//InGaAs is higher than that of GaInP/GaAs//Si, which may have resulted from the electrode thickness and wire bond resistance. Here, the electrode thickness of AuGe/Au (50/120 nm) is too thin for the InGaAs and results in the contact resistance becoming larger after the wire bond. The decrease in R_p_ with the increasing number of sub-cells could be due to the surface and cross-section increasing after the mechanical stacking and wire bonding. This parallel resistance could result in the V_oc_ reducing (140 mV for GaInP/GaAs//Si and 100 mV for GaInP/GaAs//InGaAs, respectively) after the mechanical stacking and wire bonding.

**Table 3:** Calculate series resistance (R_s_) and parallel resistance (R_p_) of the single- dual- triple-junction solar cells with various junctions based on equation (1).

Si type	R_s_ (Ω)	R_p_ (Ω)
Single-junction Si solar cell	31	380
Dual-junction solar cell	726	6000
Triple-junction solar cell	763	5423
**InGaAs type**	**R**_**s**_ **(Ω)**	**R**_**p**_ **(Ω)**
Single-junction InGaAs solar cell	42	2109
Dual-junction solar cell	647	5274
Triple-junction solar cell	983	4040

R_s_: series resistance; R_p_: parallel resistance.

**Figure 6:**
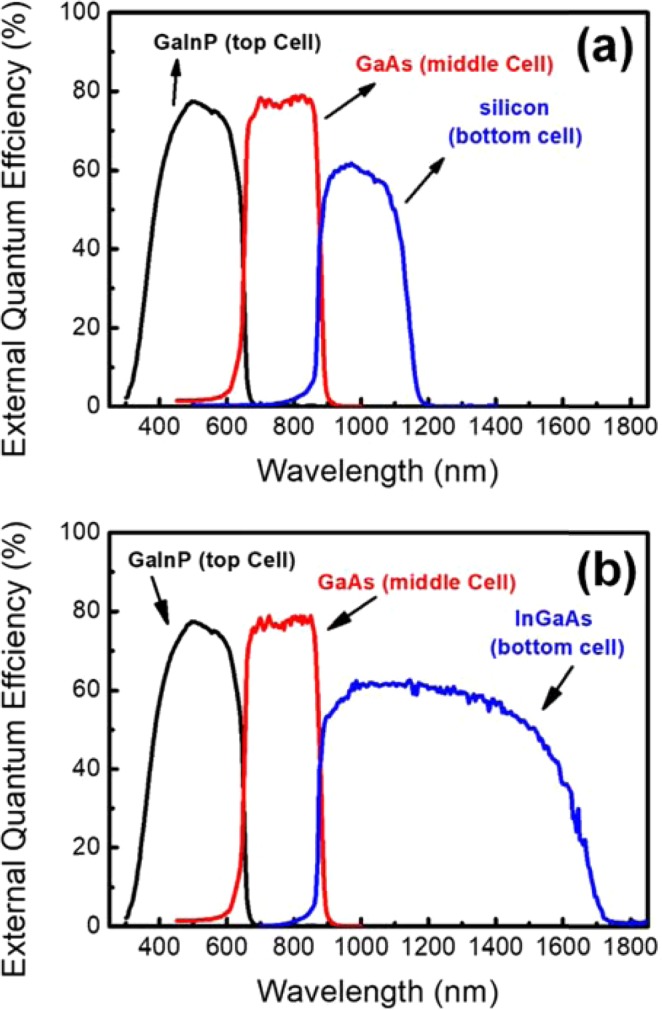
EQE characteristics of (**a**) GaInP/GaAs//Si and (**b**) GaInP/GaAs//InGaAs triple-junction solar cells measured at room temperature.

Figure 6 shows the EQE of the GaInP/GaAs//Si (Fig. 6(a)) and the GaInP/GaAs//In_0.51_Ga_0.49_As (Fig. 6(b)) solar cells in the wavelength regions of 300–1200 and 300–1800 nm, respectively. This main aim of this study was to develop a simple mechanical stacking method to fabricate the GaInP/GaAs back-side electrode layer of a DJ solar cell. As the result, the EQE of the GaInP/GaAs DJ solar cell was found to be consistent in 300–900 nm wavelength region, and values exceeding 75% can be obtained from the top and the middle cells. Wavelengths of up to 1200 nm can be absorbed by the GaInP/GaAs DJ solar cells bonded on Si solar cells. Moreover, EQE values greater than 50% can be obtained for the bottom cell in the wavelength region of 900–1050 nm. However, the EQE value gradually degrades at wavelengths greater than 1050 nm. The light absorption spectrum of In_0.51_Ga_0.49_As extends to 1800 nm, which is broader than that of Si (900–1200 nm). EQE values exceeding 50% can be obtained for In_0.51_Ga_0.49_As from the GaInP/GaAs//In_0.51_Ga_0.49_As bottom cell in the wavelength region of 900–1600 nm. Hence, more photo-generated excess carriers in the GaInP/GaAs//In_0.51_Ga_0.49_As solar cell result in a higher J_sc_, as given in Tables 2 and 3. It can be observed that the multi-junction solar cells with high short-circuit current can be attached to In_0.51_Ga_0.49_As solar cells via DJ bonding. Moreover, the EQE of In_0.51_Ga_0.49_As in the GaInP/GaAs//In_0.51_Ga_0.49_As TJ solar cell is smaller than that of Si in the GaInP/GaAs//Si TJ solar cell. Although the bottom cell in the GaInP/GaAs//In_0.51_Ga_0.49_As TJ solar cell absorbs light over a wide wavelength range, its low V_oc_ and EQE result in lower efficiency than that of GaInP/GaAs//Si TJ solar cells.

In order to understand the short-circuit current and open-circuit voltage behaviors for the TJ solar cell before and after stacking, SCAPS software was used to conduct a one-dimensional simulation. A drift-diffusion model and AM1.5 solar spectrum are used in this simulation. A J–V simulation of each subcell was performed to calculate the respective photocurrent and to ensure current-matching conditions. Table 4 lists the parameters used in the GaInP, GaAs, Si, and InGaAs subcells. All of the material parameters are taken from refs^32–36^. Because InGaAs and InP are heterojunctions, there are traps in the interface, and the interface recombination velocity is assumed to be 10^5^ cm/s in this work^37^. The radiative recombination coefficient of GaInP was taken from the literature^35^. According to refs^35,38^, the recombination velocity is 9 × 10^5^ cm/s at the InAlP and GaInP interface and 8 × 10^4^ cm/s at the AlGaInP and GaInP interface. The AM1.5 spectrum illuminates the TJ stacking, and each subcell is responsible for the absorption of the designated spectral portion, which is from its bandgap energy (E_G_) to the bandgap energy of the subcell above it. Under an ideal coupling condition using metal-wire connection, the V_oc_ will be the sum of the V_oc_ values of the subcells, and the J_sc_ will be limited to the smallest value of each of the three subcells. Ideal coupling is actually a satisfactory approximation because in this work metal wiring was utilized in the mechanically stacked tandem cell to connect the middle cell and the bottom cell.

**Table 4:** Parameters used simulation for GaInP, GaAs, Si, and InGaAs subcells.

Parameters	GaInP	GaAs	InGaAs	Si
Band gap (eV)	1.9	1.424	0.74	1.1
Electron affinity (eV)	4.16	4.07	4.49	1.05
Relative permittivity	11.6	13.2	12.5	11.9
CB effective density of state (cm^−3^)	1.3 × 10^20^	4 × 10^17^	2.1 × 10^17^	2.8 × 10^19^
VB effective density of state (cm^−3^)	1.28 × 10^19^	9 × 10^18^	7.7 × 10^18^	1.04 × 10^19^
Electron mobility (cm^2^/Vs)	500	3197	12260	1500
Hole mobility (cm^2^/Vs)	30	232	300	450
Radiative recombination coefficient (cm^3^/s)	5 × 10^−9^	1.3 × 10^−10^	1.43 × 10^−10^	1.8 × 10^−15^
Auger electron capture coefficient (cm^6^/s)	—	5 × 10^−31^	8.1 × 10^−29^	3 × 10^−31^
Auger hole capture coefficient (cm^6^/s)	—	5 × 10^−31^	8.1 × 10^−29^	3 × 10^–31^
SRH τ_n_ (ns)	—	9 × 10^2^	2 × 10^4^	4.3 × 10^3^
SRH τ_p_ (ns)	—	9 × 10^2^	2 × 10^4^	6.1 × 10^3^

The purpose of this simulation was to show that the excessive J_sc_ of the InGaAs bottom cell is not useful for providing higher efficiency, and thus examining the respective subcell’s J_sc_ in a series-connected configuration is illustrative. Table 5 lists the J_sc_ and V_oc_ of each subcell, and assuming ideal coupling, the simulation result can be compared to the experimental values. In Table 5, it can be seen that the current was limited by the middle cell (GaAs) and bottom cell (Si) in the GaInP/GaAs//InGaAs and GaInP/GaAs//Si TJ solar cells, respectively. This is because the short-circuit current of the InGaAs solar cell is higher than that of the Si solar cell. The open–circuit voltages are 2.72 V and 2.89 V for GaInP/GaAs//InGaAs and GaInP/GaAs//Si TJ solar cells, respectively, owing to the V_oc_ of the InGaAs solar cell being smaller than that of the Si solar cell. The InGaAs solar cell provides a large photocurrent, but this does not benefit higher efficiency, because of the limiting of J_sc_ by the middle GaAs cell and the small V_oc_. Obviously, the Si solar cell is a better choice because it can provide a higher V_oc_ than InGaAs and, most importantly, Si has a much lower cost than InGaAs. In addition, our drift-diffusion calculation result is consistent with earlier literature^39^, where it was shown that under a one-sun condition and using detailed balance analysis, the optimal bottom cell bandgap is ~1 eV for TJ solar cells. Thus, it can be seen from calculation that Si is indeed a better choice than InGaAs, as far as being the bottom cell of a TJ cell is concerned.

**Table 5:** Electrical properties of solar cells with various device structures obtained from simulation and experiment.

	J_sc_ (mA)/cm^2^	V_oc_ (V)
GaInP (simulation)	13.78	1.3
GaAs (simulation)	11.63	1.03
Si (simulation)	10.17	0.56
InGaAs (simulation)	25.38	0.39
GaInP/GaAs//InGaAs Triple- junction (simulation)	11.63	2.72
GaInP/GaAs//Si Triple- junction (simulation)	10.17	2.89
GaInP/GaAs//InGaAs Triple- junction (experiment)	13.66	2.52
GaInP/GaAs//Si Triple- junction (experiment)	13.37	2.71

## Conclusion

GaInP/GaAs//Si and GaInP/GaAs//InGaAs triple-junction (TJ) solar cells were successfully fabricated via mechanical stacking and wire bonding. Indium tin oxide (ITO) films possess a transparency higher than 90% for wavelengths of 880–1000 nm and a low contact resistance of 1.62 × 10^−4^ Ω-cm^2^ with respect to the p^+^-GaAs layer, confirming that they are highly suitable for use as the transparent conductive layer in solar cells. The results demonstrate that ITO films have good potential for being used as the back-side electrode in solar cells, and they have high transmittance, unlike the opacity of conventional metal materials.

According to J–V measurements, the GaInP/GaAs//Si TJ solar cell can yield an efficiency of 27.31% with a V_oc_ of 2.71 V, a J_sc_ of 13.37 mA/cm^2^, and a fill factor of 75.40%. Moreover, the conversion efficiency of the GaInP/GaAs//InGaAs multi-junction solar cell under the one-sun condition in the AM1.5 G solar simulator was 26.95% with a V_oc_ of 2.52 V, a J_sc_ of 13.66 mA/cm^2^, and an FF of 78.30%. Compared to the Si-based and the InGaAs-based dual-junction solar cells, the conversion efficiency of the TJ cell was higher by 32.6% and 30.9%, respectively. This shows that wire bonding and mechanical stacking are useful for increasing the conversion efficiency of multi-junction solar cells. From the J–V curve results, two points can be inferred. A DJ solar cell bonding with Si cell can result in a higher V_oc_, while bonding with InGaAs cell can achieve a higher short-circuit current. This study demonstrated the great potential of wire bonding and utilizing ITO as the channel for electron transport to achieve high conversion efficiency in solar cells with three or more junctions.

## Methods

**Figure 7:**
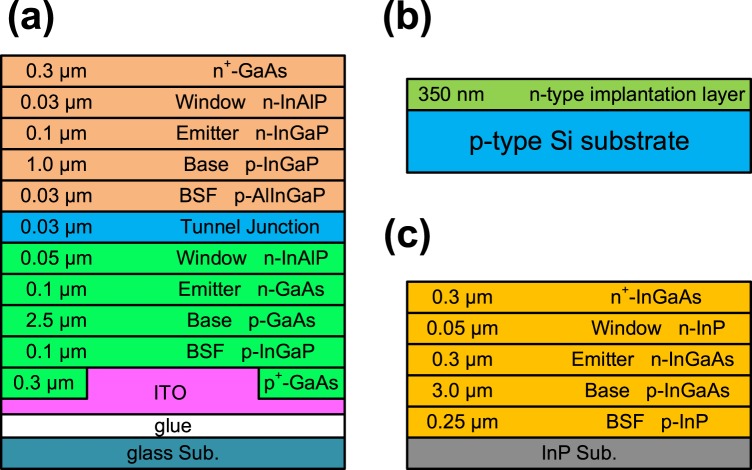
Structures of the solar cell devices with (**a**) a GaInP/GaAs DJ solar cell, (**b**) a Si solar cell, and (**c**) an InGaAs solar cell.

In this study, epitaxial structures of inverted Ga_0.51_In_0.49_P (with energy bandgap 1.9 eV)/GaAs (with energy bandgap 1.4 eV) DJ solar cells and In_0.53_Ga_0.47_As (with energy bandgap 0.75 eV) solar cells were grown on 2-in.-diameter p-type GaAs (100) wafers and 2-in.-diameter p-type InP (100) substrates, respectively, using MOVPE. The schematic structures of the solar cells are shown in Fig. 7, where Fig. 7(a) shows a GaInP/GaAs DJ solar cell, Fig. 7(b) shows a Si solar cell, and Fig. 7(c) shows an InGaAs solar cell. In the process of epitaxial growth, trimethylgallium (TMGa), trimethylindium (TMIn), arsine (AsH_3_), and phosphine (PH_3_) were used as precursors of group III and V compounds. Purified hydrogen was employed as the carrier gas. Additionally, the growth temperature was maintained at 650 °C, and the reactor pressure was set to 60 mbar. To efficiently reduce contact resistance between the electrode (indium tin oxide; ITO) and the DJ solar cell, a p^+^-GaAs contact layer was grown on the top of the DJ solar cell before ITO deposition and the bonding process. The doping concentration and thickness of the p^+^-GaAs layer was optimized to 8 × 10^18^ cm^−3^ and 300 nm, respectively. In order to reduce the p^+^-GaAs layer absorption, a dot array with the 300 μm diameter and 600 μm spacing was fabricated by wet etching.

**Figure 8:**
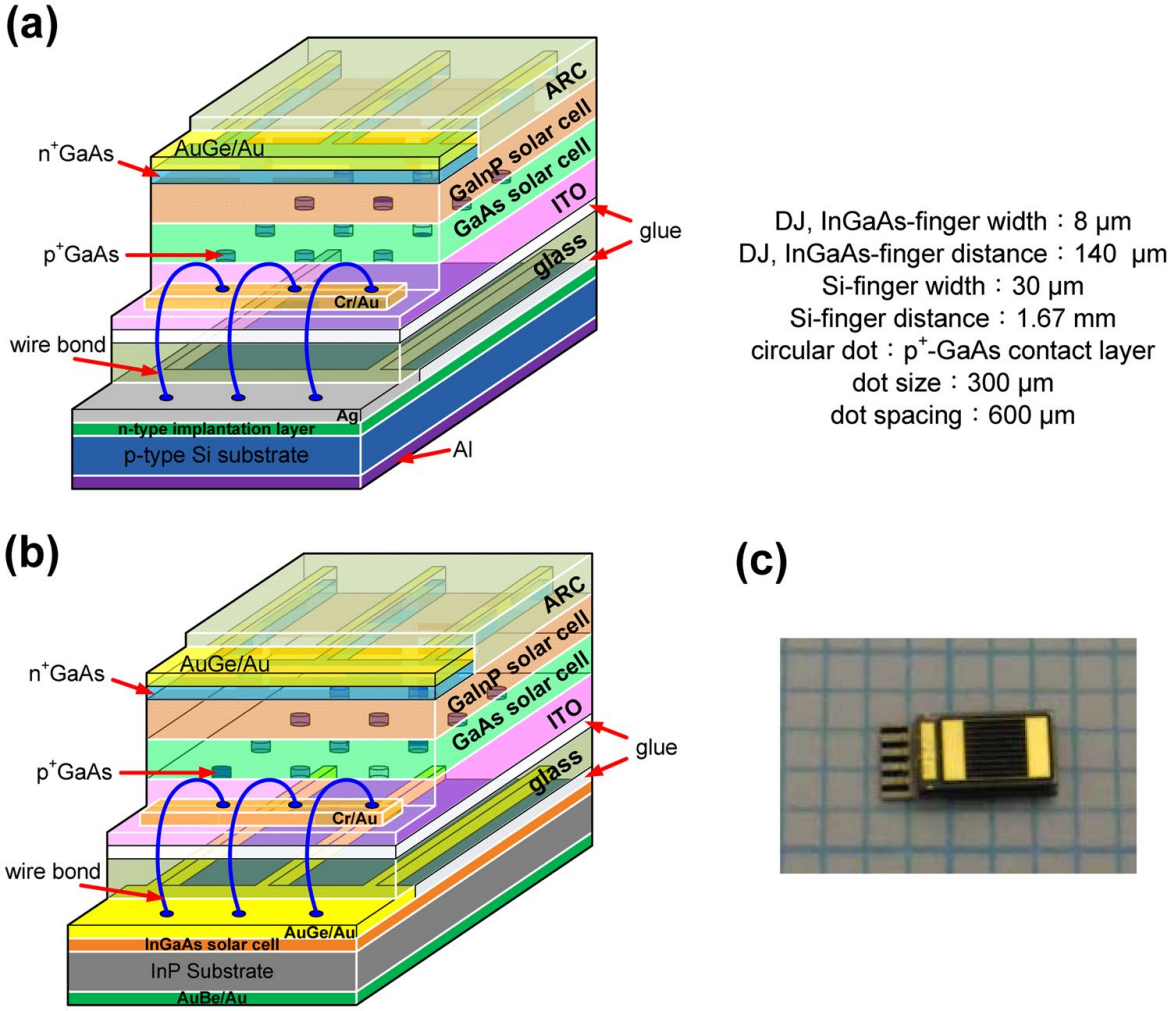
Schematic diagrams of mechanically stacked solar cells prepared with (**a**) GaInP/GaAs//Si, (**b**) GaInP/GaAs//InGaAs TJ solar cells and (**c**) photo of GaInP/GaAs//Si TJ solar cells.

The device fabrication processes were performed as following. ITO films with thickness 200 nm were used as the back-side electrodes and were deposited using an e-beam evaporator onto the p^+^-GaAs contact layer with dot array of a GaInP/GaAs DJ solar cell. For the growth of a 200-nm-thick ITO layer, the deposition temperature was maintained at 270 °C in an O_2_ atmosphere of 2 × 10^−4^ Torr. Furthermore, inverted GaInP/GaAs DJ solar cells with an ITO layer was stacked to glass by glue bonded. The glue used was silicone type and the glass used was Corning 1737 glass with high transmission of wavelengths above 350 nm. Then the GaAs substrate was removed to expose the GaInP top solar cell with an n^+^-GaAs contact layer. AuGe/Au (50/120 nm) was deposited on the n^+^-GaAs and n^+^-InGaAs contact layers of the GaInP/GaAs DJ and InGaAs solar cells for the front-side contact metal via thermal evaporation. The AuGe/Au can be Ohmic contact with n^+^-GaAs and n^+^-InGaAs contact layers without thermal annealing due to the heavily doping for these layers. The back-side contact metal of the AuBe/Au (50/120 nm) on the p-type InP substrate for cell devices was coated via thermal evaporation. The Si solar cell was fabricated using a single-crystal p-type Si substrate with a sheet resistance of 100–200 Ω/□, then implanted with PH_3_ and activated at 825 °C to obtain a top n-layer with thickness 350 nm and sheet resistance 72 Ω/□. In the case of the Si solar cell, the front-side Ni/Ag electrode (50 nm/1.5 µm) was deposited on the n-type Si devices using an e-beam evaporator, and aluminum paste was coated on the back electrode via screen printing. Subsequently, the GaInP/GaAs DJ solar cells were bonded onto silicon and InGaAs solar cells via the glue-bonding method, which uses a glue material with high-optical-transmission and mechanical bonding at low temperatures. Then, the wire-bonding method was used to connect the GaInP/GaAs metal pad, Si metal pad, and InGaAs metal pad to form the GaInP/GaAs//Si (Fig. 8(a)) and GaInP/GaAs//InGaAs (Fig. 8(b)) TJ solar cells. The wire bonded area is partial ITO deposited Cr/Au for the top DJ cell and Ag or AuGe/Au for the Si and InGaAs bottom cells, respectively. Here, the contact resistance between ITO and Cr/Au is about 5.73 × 10^−4^ Ω-cm^2^. The function of wire bonding is the same as the tunnel junction in the tandem solar cells. The chip size of DJ solar cells was 1.73 mm × 3.23 mm. The area of the bottom solar cell is the same with that of DJ solar cell. The extra area of the bottom solar cell is used for the wire bonding. Figure 8(c) shows the photo of GaInP/GaAs//Si TJ solar cells. The shadowing factor (the top metal on the GaInP solar cell) of the light-receiving region was approximately 6%.

The optical transmittance of the DJ solar cell film was measured using an N&K analyzer (model: 1280, N&K Technology). The circular transmission line model (CTLM) measurement was used to evaluate the associated specific contact resistance between the ITO film and the p^+^-GaAs contact layer. The illuminated J–V characteristics of the cell devices were investigated at room temperature using a standard solar simulator (WACOM) equipped with a one-sun AM1.5G light source (100 mW/cm^2^).

In this study, we also use simulation to predict the theoretical stacked short-circuit current density and open-circuit voltage for these kinds of solar cells and compared them with the real devices.

## Supplementary information

Supplementary Information
