# Supplementary material for: Performance comparison of III–V//Si and III–V//InGaAs multi-junction solar cells fabricated by the combination of mechanical stacking and wire bonding
Source: Sci Rep. 2019 Mar 13;9:4308. doi: 10.1038/s41598-019-40727-y (PMC6416321; doi:10.1038/s41598-019-40727-y)
Supplement: Supplementary file 1 — Supplementary Information [file 41598_2019_40727_MOESM1_ESM.docx]

**Supplementary Information**

**Performance comparison of III–V//Si and III–V//InGaAs multi-junction solar cells fabricated by the combination of mechanical stacking and wire bonding**

Yu-Cheng Kao^1^, Hao-Ming Chou^2^, Shun-Chieh Hsu^3^, Albert Lin^2^, Chien-Chung Lin^3^, Zun-Hao Shih^4^, Chun-Ling Chang^4^, Hwen-Fen Hong^4^, and Ray-Hua Horng^1,2,5^

*^1^* *Graduate Institute of Precision Engineering, National Chung Hsing University, Taichung, 40227, Taiwan, ROC.*

*^2^* *Institute of Electronics, National Chiao Tung University, Hsinchu, 30010, Taiwan, ROC.*

*^3^ Institute of Photonic System, National Chiao Tung University, Tainan, 71150, Taiwan, ROC.*

*^4^ Institute of Nuclear Energy Research (INER), Atomic Energy Council, Executive Yuan, Taoyuan, 32546, Taiwan, ROC.*

*^5^ Center for Emergent Functional Matter Science, National Chiao Tung University, Hsinchu 300, Taiwan, ROC.*

**The consideration of reflection caused by insertion of glass:**

Several cases were set up for the optical reflection simulation to understand the possible causes of the variation of photovoltaic currents in this three-junction cell.

First, we consider the case between the insertion of the bottom cell. In the case 1 and case 2, two structures are designed, as shown in Fig. S1:


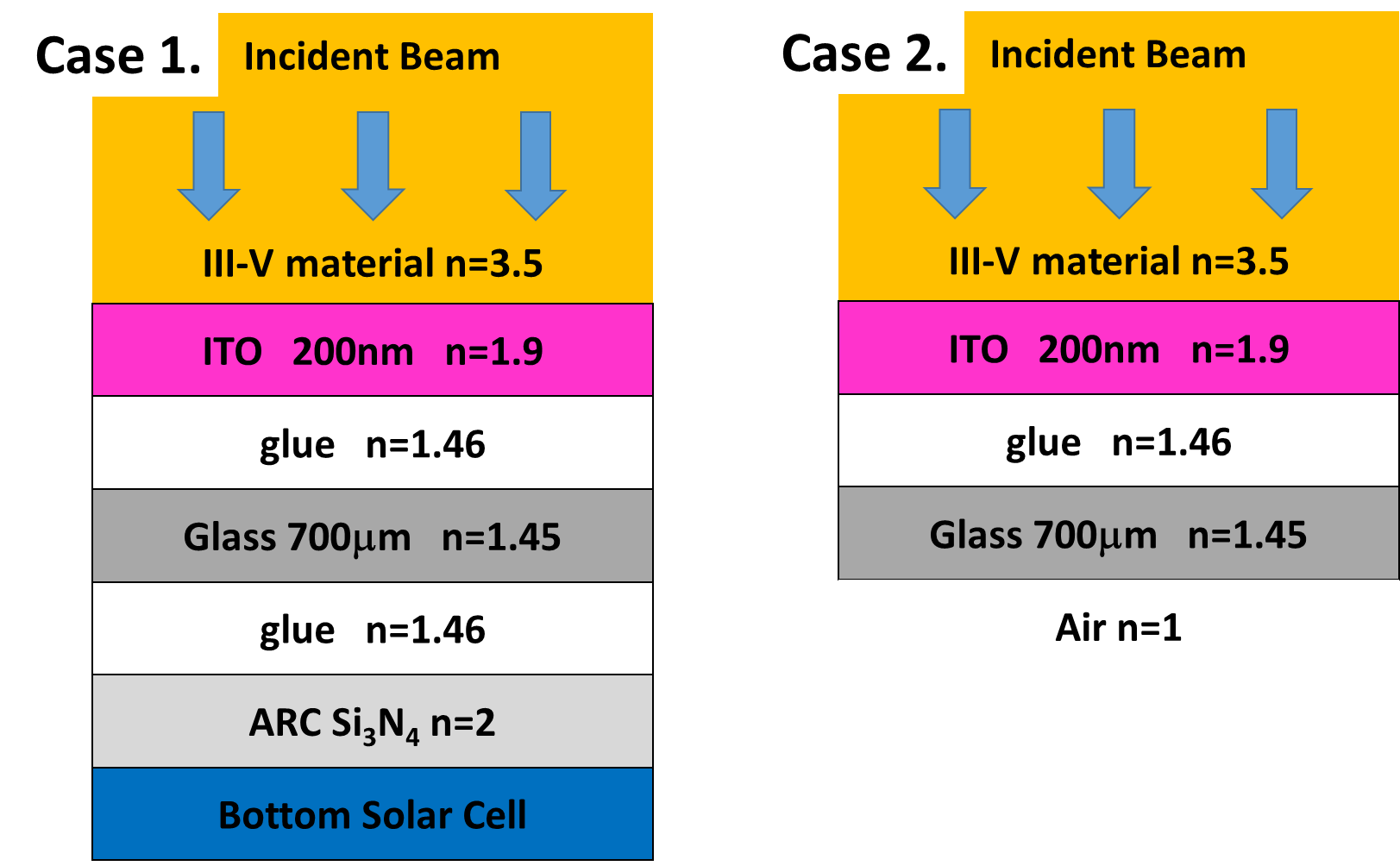


Fig. S1 Schematic diagrams for case 1 and case 2 in the simulation.

In these two cases, the difference is with (case 1) and without (case 2) the bottom cell. From the transfer matrix calculation, while it’s oscillating, if we use the averaged reflectance to smooth out these oscillations, a slightly higher reflectivity spectrum can be seen in the case 1 versus case 2, as shown in the Fig. S2. From the averaged reflection in the range of 800 nm to 1000 nm, we saw a less reflective interface for case 2 (no bottom), and more photons are reflected back to the dual-junction material when the bottom cell was added (case1). The higher reflected percentage of photons in the bottom cell could mean less photons to be absorbed in the bottom cell (especially in the 800-1000nm range), and thus a lower measured bottom junction EQE in the bottom cell can be observed in the Fig. 6 of the paper.


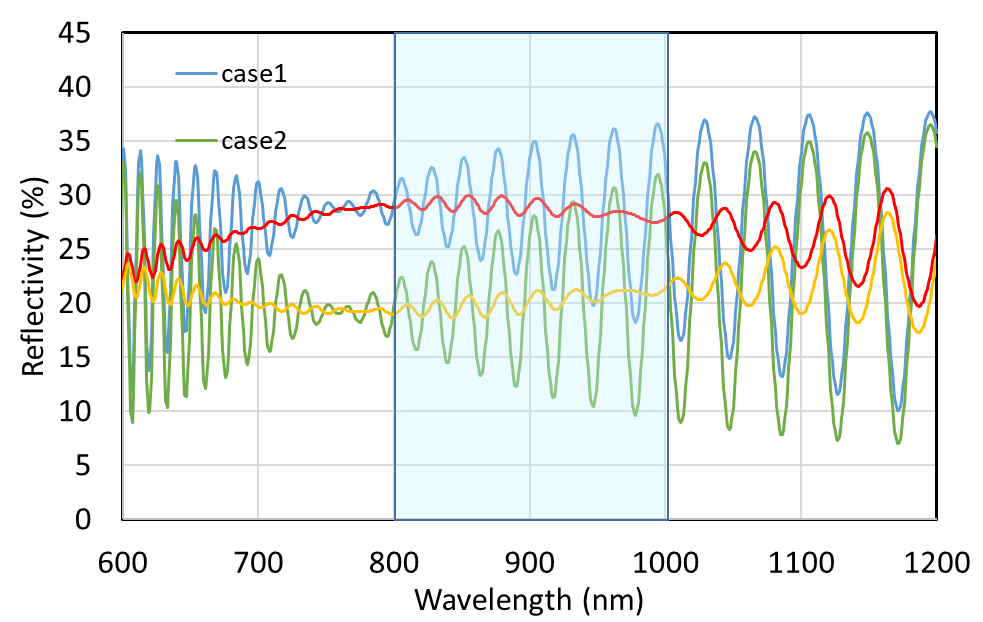


Fig. S2 Reflectivity spectra of case 1 and case 2 and the moving averaged reflectance (red and orange).

However, from the photovoltaic current-voltage measurement, we did observe the increase of the short-circuit current. The possible causes of this phenomenon can be explained as follows:

First, the thickness of the glass substrate is much larger than the dual-junction device. Normal Corning 1734 glass is 700 μm thick while the dual-junction device is only 4.24μm thick. The incident photons can thus be stored (or guided) within this giant glass substrate (called light guide in the LCD technology). Two cases are set up to evaluate this condition. The structures (named case 3 and case 4) are shown in Fig. S3:


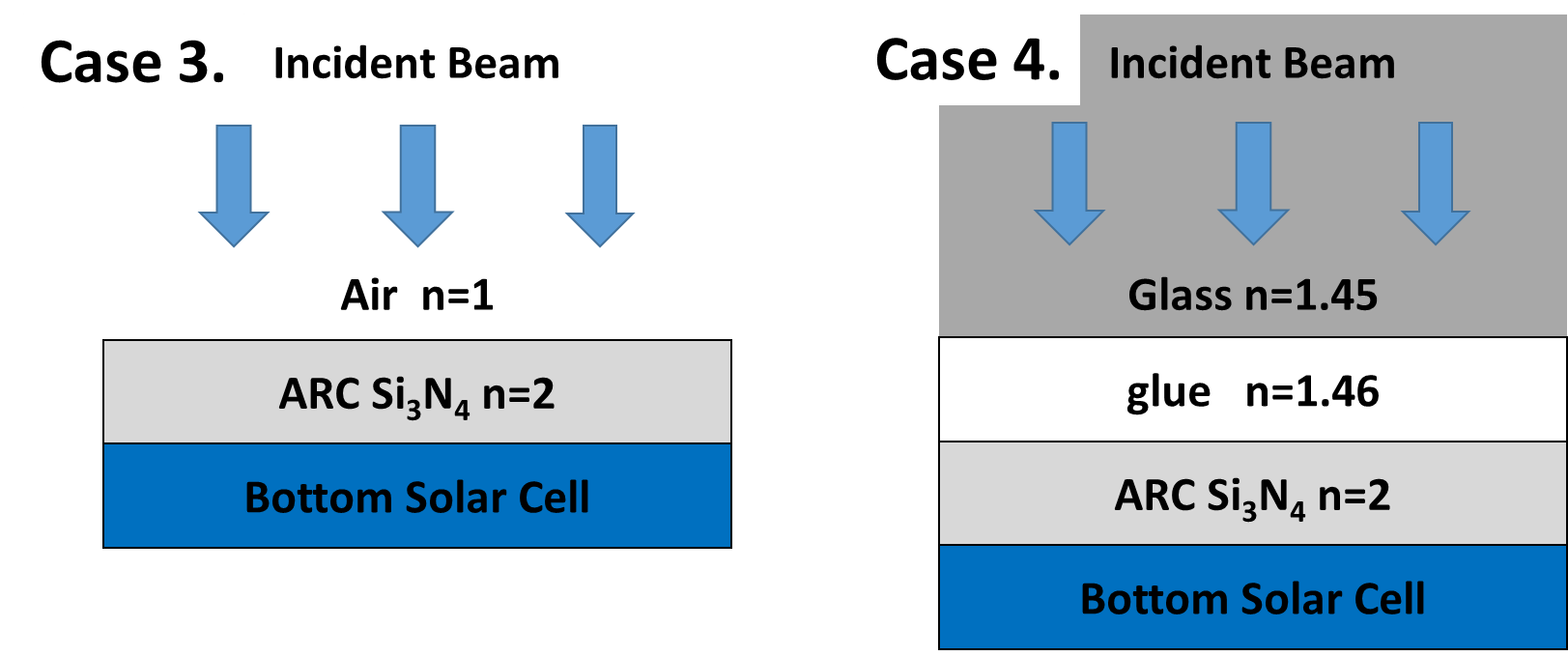


Fig. S3 Schematic diagrams of case 3 and case 4 in the simulation

From our simulation, the reflectance of the interface for glass and air as incident media can be very different (averaged 10.6% of reflection for case 3 and 6.1% for case 4, respectively). Although the device has a very good anti-reflection coating in the air for visible range, the stacked solar cell condition (case 4) can certainly gain more photons in the 800 nm to 1200 nm range. Thus it is much easier for photons to be absorbed in the bottom cell once they are into the glass substrate. So that is why the short-circuit current slightly increases after the bottom cell attached.


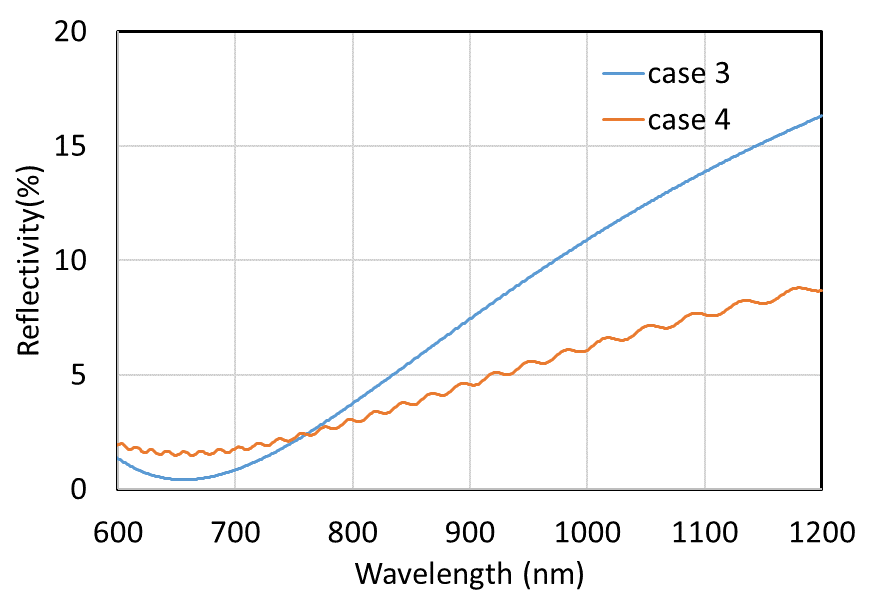


Fig. S4 Reflectance spectra for case 3 and case 4.
